# Aufklärung durch behördliche Impfkommunikation: Ein experimenteller Vergleich von evidenzbasierten Faktenboxen, Nudges und Werbung

**DOI:** 10.1007/s00103-025-04109-2

**Published:** 2025-08-04

**Authors:** Felix G. Rebitschek, Mirjam A. Jenny, Gert G. Wagner, Christin Ellermann

**Affiliations:** 1 Harding-Zentrum für Risikokompetenz, Fakultät für Gesundheitswissenschaften, Universität Potsdam, Virchowstr. 2, 14482 Potsdam, Deutschland; 2 Em. Gigerenzer, Max-Planck-Institut für Bildungsforschung, Berlin, Deutschland; 3 Institute for Planetary Health Behaviour, Gesundheitskommunikation, Universität Erfurt, Erfurt, Deutschland; 4 Forschungsgruppe Gesundheitskommunikation, Implementation, Bernhard-Nocht-Institut für Tropenmedizin, Hamburg, Deutschland; 5 Center for Adaptive Rationality, Max-Planck-Institut für Bildungsforschung, Berlin, Deutschland

**Keywords:** Informierte Entscheidung, Evidenzbasierte Gesundheitsinformationen, Evidenz-Zusammenfassungen, Risikokommunikation, Persuasive Kommunikation, Informed choice, Evidence-based health information, Evidence summaries, Risk communication, Persuasive communication

## Abstract

**Einleitung:**

Evidenzbasierte Gesundheitsinformationen als Instrument der Gesundheitsaufklärung unterstützen informierte Impfentscheidungen. Demgegenüber stehen verhaltenssteuernde Ansätze. Die Eignung verschiedener Aufklärungsansätze scheint von der anfänglichen Impfbereitschaft und den Informationsbedürfnissen (z. B. von Impfunentschiedenen, -skeptikern) abzuhängen.

**Methoden:**

Mithilfe des „Corona-Online-Meinungs-Panel-Survey-Spezial“-(COMPASS-)Befragungspanels führten wir im Mai 2021 ein präregistriertes Experiment (*N* = 2944) mit verschiedenen Impfaufklärungsansätzen durch: evidenzbasierte tabellarische und grafische Faktenbox vs. Norm- und moralischer Nudge vs. Impfwerbung. Vor und nach der Impfaufklärung wurden Impfabsichten, Verstehen der Impfung, Einstellung sowie Vertrauen in die Impfstoffe von Impfbereiten, -geneigten, -skeptischen, -gegnern und -unentschiedenen gemessen.

**Ergebnisse:**

Faktenboxen unterstützten das Verstehen der Impfung. Grafische Faktenboxen verstärkten Impfabsichten von Unentschiedenen mit Informationsbedürfnissen und Impfskeptischen. Der moralische, aber nicht der Norm-Nudge verstärkte Impfabsichten von Impfgeneigten und von -unentschiedenen ohne Informationsbedürfnisse. Keiner der Ansätze zeigte einen negativen Einfluss auf das Vertrauen in Impfstoffe.

**Diskussion:**

Evidenzbasierte Informationen wirken dem gesellschaftlichen Ziel einer hohen Durchimpfung der Bevölkerung nicht entgegen. Zielgruppen mit verschiedenen Impfabsichten und Informationsbedarfen reagieren unterschiedlich auf Ansätze der Impfaufklärung bzw. -steuerung. Bei Nudging und Impfwerbung sollten Fragen ethischer und rechtlicher Verantwortung diskutiert werden.

**Zusatzmaterial online:**

Zusätzliche Informationen sind in der Online-Version dieses Artikels (10.1007/s00103-025-04109-2) enthalten.

## Abstract (en)

**Introduction:**

Evidence-based health information is a key tool in health communication to support informed decision-making. In contrast, there are behavioural approaches. The suitability of the different approaches seems to depend on the initial willingness to vaccinate and the information needs (e.g. of undecided people, sceptics).

**Methods:**

Using the longitudinal Corona Online Opinion Panel Survey Special (COMPASS) survey panel, we conducted a preregistered experiment (*N* = 2944) in May 2021 involving information presentations on vaccination: evidence-based tabular and graphical fact box vs. norm and moral nudge vs. vaccination advertising. Before and after vaccination education, vaccination intentions, understanding of vaccination, and attitudes toward and trust in vaccines were measured among those willing, inclined, sceptical, opposed and undecided.

**Results:**

Fact boxes supported understanding of vaccination. Graphical fact boxes reinforced vaccination intention of undecideds without information needs. The moral nudge, but not the norm nudge, increased the vaccination intention of vaccination sceptics and undecided people without information needs. No negative influence on trust in vaccines could be identified.

**Discussion:**

Evidence-based information does not counteract the social goal of high immunisation coverage of the population. Depending on the intention to vaccinate and the need for information, different target groups react differently to vaccination communication. Questions of ethical and legal responsibility should be discussed with regard to nudging and vaccination advertising.

## Einleitung

In Gesundheitssystemen mit ausreichender Impfstoffverfügbarkeit besteht innerhalb der Bevölkerung häufig eine Zurückhaltung gegenüber verschiedenen Impfstoffen (Vaccination Hesitancy). Infolgedessen erkranken Menschen an eigentlich vermeidbaren Infektionskrankheiten und solchen, die bereits eliminiert waren oder kurz davorstanden. Zudem können besonders vulnerable Gruppen nicht mehr ausreichend von der Gemeinschaftsimmunität profitieren [1]. Ein Beispiel hierfür bildet die Covid-19-Immunisierung: In den Organisation für wissenschaftliche Zusammenarbeit und Entwicklung(OECD)-Ländern, darunter Deutschland, die USA und Frankreich, war eine „Impfmüdigkeit“ von bis zu 20 % zu verzeichnen, die sich mit zunehmender Verfügbarkeit der Impfstoffe noch verstärkte [2]. Angesichts dieser Entwicklung und mit Blick auf zukünftige Pandemien hat diese Arbeit das Ziel, evidenzbasierte Erkenntnisse zur Verbesserung behördlicher Impfstrategien zu gewinnen.

Systemisches Misstrauen [3] und Furcht gegenüber Impfstoffen bzw. ihren Nebenwirkungen – insbesondere im Zusammenhang mit seltenen, aber schwerwiegenden Ereignissen wie den Sinusvenenthrombosen nach der Covid-19-Impfung mit dem AstraZeneca-Impfstoff – können Impfabsichten und Einstellungen gegenüber Impfungen negativ beeinflussen. Potenzielle Auswirkungen zeigen sich laut Robert Koch-Institut (RKI) in einem unzureichenden Impfschutz über alle Altersgruppen, in bestehenden Impflücken (z. B. bei Grippeimpfungen), stagnierenden Impfquoten (z. B. bei der HPV-Impfung bei Jugendlichen) sowie in verspätet oder gar nicht abgeschlossenen Impfserien (z. B. bei Kleinkindern; [4]). Auch gesundheitspolitisches Handeln, bestehende Unsicherheiten bezüglich der impfbezogenen Evidenzlage sowie die „Infodemie“ [1] – insbesondere durch die rasche Verbreitung von Falsch- und irreführenden Informationen – können die Impfbereitschaft beeinträchtigen. Determinanten der Impfbereitschaft lassen sich grundsätzlich durch das 5C-Modell [5] bzw. in seiner Erweiterung „7C“ [6] beschreiben, messen und als Zielgrößen für die Erarbeitung von nationalen Impfstrategien berücksichtigen:

1. Confidence: Vertrauen in die Impfung erhalten und fördern,
2. Complacency: Risikowahrnehmung bezüglich der zu vermeidenden Erkrankung schärfen,
3. Constraints: Barrieren in der Zugänglichkeit zum Impfstoff senken,
4. Calculation: geeignete Suche nach entscheidungsrelevanten Informationen ermöglichen,
5. Collective Responsibility: Verantwortungsgefühl für die Gemeinschaft stärken,
6. Compliance: Regelkonformität unterstützen,
7. Conspiracy: Verschwörungsüberzeugungen entgegenwirken.

Aus politisch-behördlicher Sicht auf den Bevölkerungsschutz, der darauf abzielt, möglichst hohe Impfraten in der Bevölkerung zum Schutz aller bzw. einzelner (vulnerabler) Gruppen zu erreichen [7], stellt sich die Frage, wie eine hohe Impfbereitschaft erreicht werden kann, ohne die Autonomie und das Recht des Einzelnen auf Unversehrtheit zu beeinträchtigen [8]. Hier lassen sich verschiedene Ansätze und Strategien identifizieren, die von Verhaltenssteuerung bis hin zu einer rein aufklärenden Impfkommunikation reichen [9] und deren Vor- und Nachteile es zu berücksichtigen gilt: Impfakzeptanz lässt sich zum Beispiel durch unmittelbaren individuellen Nutzen, wie freie Tage nach der Impfung, finanzielle Anreize [10] und Steuervorteile, stimulieren [11]. Eine finanzielle Entschädigung für die Impfung könnte jedoch zu einem Verlust der intrinsischen Motivation führen und die Impfbereitschaft auch beeinträchtigen, wenn Menschen den Eindruck gewinnen, dass eine Impfung ohne Entschädigung nicht zu empfehlen ist [12, 13].

Das Spektrum politischer Instrumente mit Verhaltenswirkung – neben Bildung und Aufklärungskampagnen – reicht von Gesetzen und Vorschriften (z. B. Tätigkeitsverboten nach § 20a Infektionsschutzgesetz im Jahr 2022 [14]), einschließlich Zwangsmitteln zu ihrer Durchsetzung, über Anreize [15] und Werbung bis hin zum „Anstupsen“ (Nudging; [16]). Diese Instrumente können Druck (z. B. Erwartungsdruck), sich impfen zu lassen, auf einzelne Bürger:innen innerhalb einer demokratischen Gesellschaft ausüben und bzw. oder als Druck wahrgenommen werden. Werden solche Instrumente im Rahmen einer Kampagne ohne transparente und ausgewogene Darstellungen von Nutzen und Schaden eingesetzt, kann das Vertrauen in eine Impfung geschwächt werden, was wiederum nichtintendierte Impfzurückhaltung provozieren könnte [17–19]. Zudem trüge eine solche Kampagne nicht dem Public-Health-ethischen Prinzip der Gewährleistung der Autonomie des Einzelnen [8] und der im Patientenrechtegesetz verankerten umfassenden Aufklärungspflicht Rechnung [20]. Autonomie konstituiert sich aus der kritischen Reflexion der Konsequenzen von Handlungen und Unterlassungen für das Individuum selbst und andere und ermöglicht eigenständiges Handeln [8].

Um Vertrauen in die Gesundheitspolitik und die Gesundheitssysteme für Krisensituationen aufrechtzuerhalten bzw. auch zurückzugewinnen sowie vor dem Hintergrund gesundheitlicher Gerechtigkeit, sollten gesundheitspolitische Ansätze und Strategien Bürger*innen zu autonomen Handeln befähigen [1, 8]. Hierfür ist transparente und wissenschaftlich fundierte Aufklärung zentral, gerade auch für Bürger*innen außerhalb von institutionellen Bildungsangeboten (Schulen, Berufsschulen, Volkshochschulen, Universitäten). Das Konzept des *Boosting* schlägt verhaltenswissenschaftlich fundierte, kompetenzfördernde Instrumente vor, um die Handlungsfähigkeit von Individuen zu stärken [21]. Diese zielen auf kognitive Prozesse der Entscheidenden ab und unterstützen sie dabei, ihre Entscheidungsumwelt zu gestalten.

Die informierte Entscheidungsfindung ist ethischer Standard in der Gesundheitsversorgung westlicher Gesundheitssysteme, gemäß welchem Patient*innen in die Lage versetzt werden sollen, potenzielle Vorteile und Nachteile medizinischer Handlungsoptionen abzuwägen und eine autonome Entscheidung zu treffen, für die individuelle Werte und Präferenzen berücksichtigt werden [22]. Zu diesem Zweck bedarf es einer evidenzbasierten Risikokommunikation, also einer transparenten, ausgewogenen und verständlichen Darstellung der Erkrankung, ihrer Diagnose- und Behandlungsmöglichkeiten sowie der jeweils damit verbundenen Nutzen und Risiken. Sollte dadurch eine informierte Entscheidungsfindung zum Regelfall werden, ist mit einer Vielfalt individueller Entscheidungen zu rechnen – sowohl im Sinne einer bewussten Zustimmung zur Impfung als auch eines reflektierten Verzichts. Betrachtet man alle individuellen Entscheidungskonsequenzen zusammen, hängt der gesellschaftliche Nutzen davon ab, ob hinreichend viele informierte Entscheidungen zum Impfen führen, was wiederum stark vom Nutzen-Schaden-Verhältnis beeinflusst wird.

Zwei Arten von evidenzbasierten Boosting-Formaten zur Risikokommunikation sind tabellarische und grafische Faktenboxen. Diese wurden vielfach erforscht und u. a. vom Robert Koch-Institut (RKI) 2021 zur Corona-Impfaufklärung eingesetzt. Faktenboxen sind tabellarische oder grafische Darstellungen [23], die Nutzen und Schaden medizinischer Optionen und deren Eintrittswahrscheinlichkeit transparent und ausgewogen zusammenfassen. Entscheidend ist dabei, dass die Darstellung keine Unterschiede in der Zugänglichkeit oder der Gewichtung von Nutzen und Schaden oder von Handlungsoptionen aufweist. Dadurch wird vermieden, die Rezipient:innen in eine bestimmte Entscheidungsrichtung zu lenken, wie es etwa beim Nudging der Fall wäre. Faktenboxen informieren über verschiedene Entscheidungsoptionen, darunter über medizinische Behandlungen, Krebsvorsorgeuntersuchungen und Impfungen [24–26]. Sie sollen die Menschen in die Lage versetzen, entsprechend ihren Überlegungen, persönlichen Bedürfnissen und Zielen zu handeln, und zwar auf der Grundlage der besten verfügbaren Erkenntnisse [27], aber ohne direktiv zu sein. Sie sind damit entgegen der Interpretation von einzelnen Forschenden kein Instrument zur Verhaltenssteuerung [28], sondern können als Elemente von evidenzbasierten Entscheidungshilfen (Decision Aids) eingesetzt werden. Experimentelle Studien haben gezeigt, dass Faktenboxen die Risikowahrnehmung (z. B. über Krankheitsrisiken) verbessern, verständlich sind, das Wissen über kurzfristige Risiken erhöhen und die subjektive Bewertung des Nutzen-Schaden-Verhältnisses von Impfstoffen verbessern [29–32].

Informationsmangel [3] und Informationsbedürfnisse [33] stellen zentrale Einflussfaktoren für die Impfbereitschaft dar und sind Ansatzpunkte von Kommunikationsinterventionen. Wie bei jeder Form der Kommunikation variieren jedoch mögliche Wirkungen von Risikokommunikation je nach Zielgruppe (z. B. auf Verständlichkeit, Wissenszuwachs, aber auch auf Einstellungen und Vertrauen). Dies verdeutlicht die besonderen Herausforderungen, die mit Impfaufklärung und -kommunikation einhergehen.

Mit Blick auf Impfbereitschaft und -zögerlichkeit lassen sich zunächst klar unterscheidbare Gruppen identifizieren, die bereits eine Präferenz gebildet haben (z. B. Impfbereite, Impfgegner*innen – im Extremfall klassifiziert als „accept all“, „refuse all“ [34]). Eine Gruppe besteht aus Menschen – beispielsweise mit hoher Gesundheitskompetenz [35] –, die qualitätsgesicherte Informationen über Entscheidungsoptionen aktiv suchen, finden, kritisch reflektieren und für sich nutzen. Sie haben die objektiven Informationsbedarfe für eine Impfentscheidung bereits gedeckt und sind in der Lage, eine informierte Entscheidung zu treffen, vorausgesetzt, ihre wissensbasierten Präferenzen stimmen mit der tatsächlichen Entscheidung überein. Dies wäre etwa der Fall, wenn eine positive Einstellung gegenüber der Impfung vorliegt und sich die Person nach entsprechender Aufklärung für die Impfung entscheidet. Demgegenüber gibt es jene, die bereits eine klare Position für oder gegen die Impfung bezogen haben, jedoch nicht notwendigerweise wissensbasiert. Sie könnten z. B. an falschen Überzeugungen anstelle von Faktenwissen festhalten (z. B. Impfgegner*innen, die fälschlicherweise überzeugt sind, dass eine Masernimpfung zu Autismus führt). Die Motivlage für eine solche klare Position kann aber auch eine ganz andere sein (für das Beispiel Covid-19-Impfung eine Übersicht: [36]).

In Studien zu Impfintentionen bzw. Impfbereitschaft wird im Regelfall über die Kategorien „Impfbereite“ und „Impfgegner:innen“ hinaus eine weitere Differenzierung vorgenommen [30, 36–38]. Unterschieden werden dabei häufig eher Geneigte („eher ja“), Unsichere bzw. Unentschiedene sowie Zögerliche („eher nein“) bzw. Skeptische, oft auch entlang von Rating-Skalen, abgebildet. Menschen mit einer gewissen Skepsis oder ausgeprägtem Zögern sind nicht mehr völlig unentschieden – sie haben sich bereits mit Informationen auseinandergesetzt, die ihre Absicht bzw. Bereitschaft beeinflusst und erste Überzeugungen gestützt haben, ohne dass eine abschließende Entscheidung getroffen wurde. Da somit auch keine informierte Absicht vorliegt, stellen Zögerliche bzw. Skeptische eine relevante Zielgruppe für evidenzbasierte Gesundheitsinformationen [39] bzw. Faktenboxen [32, 40] dar, die helfen können, Entscheidungskonflikte zu reduzieren.

Von besonderer Relevanz für behördliche Risikokommunikation sind zudem die Unentschiedenen, da vorhandene (auch falsche) Überzeugungen zum Impfen bislang nicht ausreichen, um eine Position einzunehmen. Grundsätzlich ist es leichter, durch Kommunikation Informationsbedürfnisse und -bedarfe zu adressieren und bei der Bildung von Überzeugungen zu unterstützen als bestehende Überzeugungen zu verändern, vor allem, wenn kein Informationsbedarf mehr wahrgenommen wird. Deshalb unterscheiden wir jene Unentschiedenen, deren Informationsbedürfnisse bislang überhaupt noch nicht erfüllt sind (z. B. aufgrund mangelnder Gesundheitskompetenz), von jenen, die keine Bedürfnisse mehr haben – ganz unabhängig von der Frage, dass sie entscheidungsrelevante Bedarfe haben mögen, die ihnen nicht bewusst sind.

Unsere erste präregistrierte Hypothese lautet: *Die Präsentation von evidenzbasierten Faktenboxen gegenüber Unentschiedenen mit Informationsbedürfnissen und skeptischen Personen der Allgemeinbevölkerung in Deutschland verbessert nicht nur das Verstehen der Impfung, sondern verringert auch die Impfzurückhaltung bei der Covid-19-Impfung****.***

Dies hätte weitreichende Implikationen für die Erreichung von Impfzielen auf Bevölkerungsebene. Zum einen würde sich das Kommunikationsmittel der Faktenbox für die genannten Zielgruppen als sinnvoller Baustein einer zukünftigen Impfstrategie empfehlen. Zum anderen würde vor dem Hintergrund Public-Health-ethischer Überlegungen aufgezeigt, dass Strategien bzw. Ansätze zur Förderung von Autonomie und informierter Entscheidungsfindung nicht im Widerspruch zu einer übergeordneten gesellschaftlichen Zielsetzung hoher Impfraten stehen müssen.

Ebenfalls zu betrachten sind Unentschiedene, die sich nicht informieren möchten und ggf. keine eigene Entscheidung treffen wollen, und jene, die eher mit einer allgemeinen Tendenz (im Pandemiefall „zu impfen“) mitgehen, ohne überzeugt zu sein. Jene Gruppen sind ebenfalls von Bedeutung für das Erreichen nationaler Impfziele. Wenn hier keine Informationsbedürfnisse vorliegen, kommen verhaltenssteuernde Methoden als theoretisch denkbare, ethisch zu reflektierende Alternativen in Betracht. Ohne die Entscheidungsoptionen für die Betroffenen einzuschränken, wäre Nudging ein möglicher Ansatz. Dabei handelt es sich um eine direktive Präsentation zugunsten einer Option (z. B. Impfen), die kognitive und emotionale Eigenschaften des Menschen gezielt nutzt bzw. ausnutzt. Nudges können sehr unterschiedlich gestaltet sein, etwa als Erinnerung an Impftermine (Reminders; [16, 41]) oder durch die Hervorhebung eines wissenschaftlichen Konsenses [42].

Wenn ein Mensch entscheidet, bewertet er oder sie Entscheidungsoptionen immer auch mithilfe von Vergleichen. Soziale Nudges nutzen diese Tendenz gezielt, etwa durch *Social Framings*, die sich auf soziale Normen beziehen [43]. Sie verdeutlichen einer Person ihren eigenen Status bzw. die eigene Handlungsposition innerhalb einer sozialen Bezugsgruppe, beispielsweise durch Hinweise, wie sich Nachbar:innen verhalten würden [44, 45]. Auch die Beeinflussung durch Peer-Verhalten kann als sozialer Nudge wirken, allerdings führt dies nicht immer zu einer höheren Akzeptanz von Impfungen [46]. Der Vergleich der eigenen Position kann aber auch mit anderen Arten von Normen angestellt werden, z. B.: „Ist meine Entscheidung gesetzeskonform?“ „Wie loyal ist das Angestelltenverhalten im Sinne der Unternehmensrichtlinien?“

Außerdem kann Nudging mit moralischem Druck verbunden werden („moral nudges“, [47]), oft mit emotionaler Ansprache (z. B. „Wollen Sie Ihre Tochter vor Krebs schützen?“; [48]), durch Hervorhebung von Solidarität, prosozialem Verhalten [49] oder Verantwortung [50, 51]. Hier wird man veranlasst, das eigene Verhalten als moralisch relevant zu empfinden, indem z. B. das Unterlassen einer Impfung als Bedrohung für die Gemeinschaft vermittelt wird. Theoretisch könnten alle Menschen durch Nudges angesprochen werden, jedoch sind Bumerangeffekte möglich [52], insbesondere bei Gruppen, die Aufklärung deutlich gegenüber Druck oder Überredung bevorzugen.

Wir stellten eine zweite präregistrierte Hypothese auf: *Nudging verringert das Zögern bei der Covid-19-Impfung bei unentschiedenen Personen ohne Informationsbedarf und jenen, die sich lieber impfen lassen würden.*

## Methoden

### Stichprobe.

**Abb. 1:**
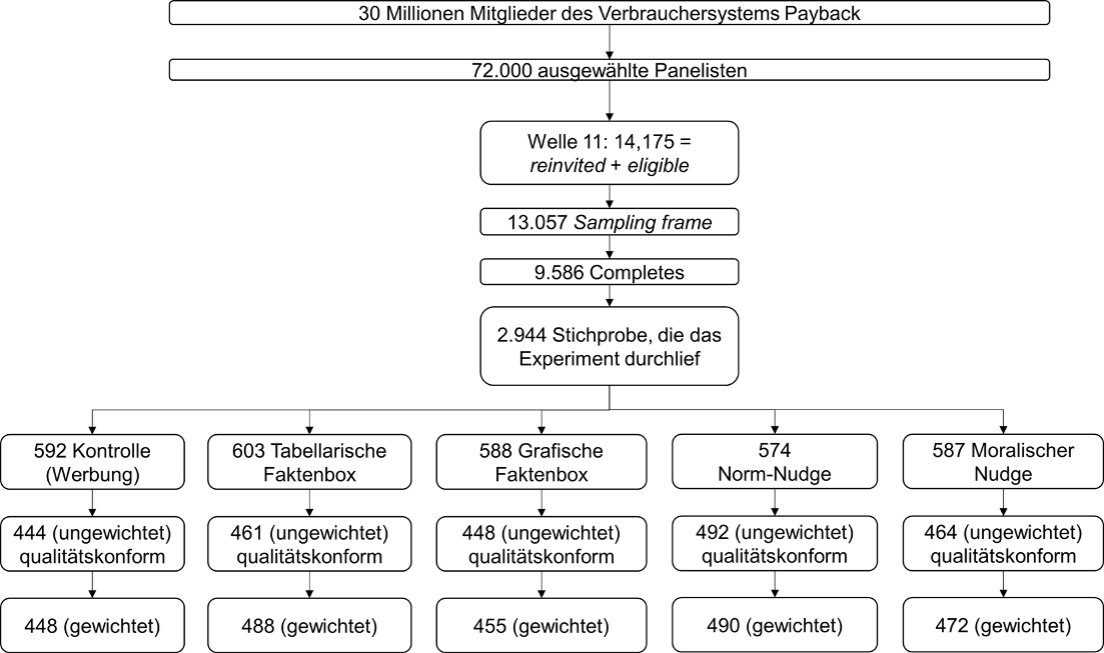
Übersicht über die Stichproben

Diese Studie nutzte die längsschnittlich angelegte Mehrthemenbefragung, das „Corona-Online-Meinungs-Panel-Survey-Spezial“ (COMPASS) von infratest dimap, welches von März 2020 an täglich 250 bis 350 Personen, die in Deutschland wahlberechtigt sind, auf Basis einer Zufallsstichprobe innerhalb des sogenannten Payback-Panels (30 Mio. Mitglieder) befragte, um pandemiebedingte Entwicklungen zu verfolgen [53]. Rund 13.000 deutschsprachige Panelist:innen bildeten hier einen mehrfach geschichteten Stichprobenrahmen aus einem Pool von insgesamt 72.000 Panelist:innen – repräsentativ für erwachsene Personen mit deutscher Staatsbürgerschaft, die online aktiv sind (Abb. 1). Diese wurden angeschrieben, wobei unter den Respondern eine einfache Zufallsstichprobe (*N* = 2944) gezogen wurde, welche auf unser Experiment gelenkt wurde. Um potenzielle Verzerrungen durch Unterschiede in der Teilnahmebereitschaft verschiedener Gruppen zu berücksichtigen (Non-Response-Bias), wurden die gezogenen Stichproben anhand demografischer Merkmale gewichtet („individual redress weighted adjustment“; mit Gewichten von 0,31 bis 3,65).

Wir führten vom 30.04. bis 18.05.2021 (Erhebungszeitraum) ein Experiment mit der Welle 11 des COMPASS-Panels durch (Abb. 1). Die ungewichtete (gewichtete) Stichprobengröße betrug *N* = 2309 (*N* = 2354). Das Durchschnittsalter der gewichteten Stichprobe von Teilnehmenden, die den Instruktionen qualitätskonform folgten, betrug 47,4 Jahre (SD = 15,0), 49,9 % waren weiblich, 0,4 % besaßen keinen bzw. noch keinen formalen Schulabschluss, 12,1 % einen Haupt‑/Volksschulabschluss, 46,4 % die mittlere Reife und 41,0 % die Hochschulreife. 24,9 % lebten allein in ihrem Haushalt. 14,0 % waren privat (voll) krankenversichert. Unter jenen, die das monatliche Haushaltsnettoeinkommen angaben, hatten 27,3 % unter 2000 €, 48,2 % zwischen 2000 € und 4000 € und 24,5 % mehr als 4000 € zur Verfügung.

### Design.

Unser präregistriertes Experiment^1^ folgte einem Mixed-5 × 2-Design mit 2 Messzeitpunkten (vor und nach der Präsentation von Informationen). Es wurden 5 Bedingungen der Informationspräsentation realisiert (Between-Subjects-Design; die Teilnehmenden wurden zufällig zugeteilt; siehe Abb. S1–S5):

1. eine Kontrollbedingung: Werbeanzeige der Bundesregierung („Ärmel hoch“),
2. eine tabellarische Faktenbox des RKI, in Abhängigkeit vom Alter unter/ab 60 Jahren,
3. eine grafische Faktenbox des RKI, in Abhängigkeit vom Alter unter/ab 60 Jahren,
4. eine Norm-Nudge-Bedingung („Freiheiten für Geimpfte“) sowie
5. eine moralische Nudge-Bedingung („andere schützen“).

### Vorgehen.

Innerhalb der 11. Erhebungswelle dieser Mehrthemenbefragung wurden erst demografische Angaben und weitere Fragen (z. B. Einstellung zur Pandemiepolitik) präsentiert, die hier nicht berichtet werden. Unser Experiment war gegen Ende der Umfrage eingesetzt und dauerte 10 min. Nach der anfänglichen Messung der Covid-19-bezogenen Impfabsicht, der Einstellung und des Vertrauens in die Impfstoffe wurde den Teilnehmenden eine Intervention (Faktenboxen oder Nudges) oder die Kontrollbedingung (Werbeanzeige) präsentiert und zugleich Verständnisfragen gestellt. Um die Wirksamkeit der Intervention zu unterstützen, konnten die jeweiligen Präsentationen nicht vor Ablauf von 60 s beendet werden. Abschließend wurden die Covid-19-bezogene Impfabsicht, die Einstellung und das Vertrauen in die Impfstoffe erneut gemessen.

### Materialien der Interventions- und Kontrollbedingungen.

Die eingesetzten Informationspräsentationen sind den Anlagen zu entnehmen (Abb. 1 und Abb. S1–S4 im Onlinematerial): Die tabellarische und die grafische Faktenbox waren im Mai 2021 im Rahmen einer Kooperation des RKI mit dem Harding-Zentrum für Risikokompetenz, Universität Potsdam, implementiert worden. Sie waren Weiterentwicklungen infolge einer ersten experimentellen Evaluation der Ursprungsversionen [30], die bereits Ende Januar 2021 (z. B. bei rki.de) veröffentlicht worden waren, und zugleich Ergebnis eines Pilotierungsprozesses. Den beiden Nudge-Texten wurden jeweils existierende Kommentare aus deutschen Tageszeitungen zugrunde gelegt, gekürzt und vereinfacht. Der Norm-Nudge stellte dabei die regulatorisch geplante größere Freiheit von geimpften Personen im Alltag in den Mittelpunkt. Der moralische Nudge bewertete das Element des verantwortungslosen Handelns.

### Messinstrumente.

Unser Fragebogen ist im Onlinematerial in Tabelle S1 zu finden. Die *Impfabsicht* wurde mit folgenden Antwortoptionen ermittelt: „Ja, sicher“, „Ja, wahrscheinlich“, „Kann ich noch nicht einschätzen/bin noch unentschieden“, „Nein, wahrscheinlich nicht“, „Nein, ganz sicher nicht“, „Ich bin schon geimpft“.

Die *Einstellung gegenüber den Impfstoffen* wurde operationalisiert, indem die Teilnehmenden aufgefordert wurden, das Nutzen-Schaden-Verhältnis der im April 2021 am breitesten verfügbaren Covid-19-Impfung (mRNA-Impfung) auf einer 11-stufigen Bewertungsskala von 0 (Schaden überwiegt eindeutig den Nutzen) bis 10 (Nutzen überwiegt eindeutig den Schaden) zu bewerten. Auch das *Vertrauen in den mRNA-Impfstoff* wurde anhand einer 11-stufigen Bewertungsskala von 0 (überhaupt kein Vertrauen) bis 10 (völliges Vertrauen) bewertet. Explorativ wurden zusätzlich die Einstellung und das Vertrauen bzgl. Vektorimpfstoffen erhoben. Abweichend von der Präregistrierung wurde der Fragebogen um das Item zum Vertrauen in das RKI gekürzt, da die Bearbeitungszeit im Pretest als zu hoch eingeschätzt wurde. Das Item wurde niedriger priorisiert, da die Kommunikatoren der Vergleichsbedingungen nicht vergleichbar waren.

*Informationsbedürfnisse* bezüglich der Impfung wurden mit einem Single-Choice-Item spezifiziert, bei dem die Teilnehmenden angeben konnten, ob sie mehr über die Wirksamkeit, die Sicherheit, damit verbundene Unsicherheiten oder gar nichts davon wissen wollten.

Wie groß das *Verständnis bezüglich des mRNA-Impfstoffes* ist, wurde mithilfe eines Items mit 5, nach dem Stand der besten verfügbaren Evidenz im April 2021 wahren und falschen Aussagen getestet: einer Aussage zum Nutzen der Impfung („Das Risiko, durch den Kontakt mit dem Coronavirus an einer schweren Covid-19-Erkrankung zu erkranken, wird um einen Faktor von etwa 10/20 [altersabhängig] reduziert” (richtig)), 2 Aussagen zum Schaden („Das Risiko, in den Tagen nach einer Impfdosis zu erschöpft für den Alltag zu sein, steigt deutlich an“ (richtig) bzw. „Es besteht ein Risiko von 1 % (10 von 1000 Personen), durch die Impfung einen schweren gesundheitlichen Schaden zu erleiden“ (falsch)) sowie 3 Aussagen zur Unsicherheit („Es ist unsicher, ob die Impfung Spätfolgen hat“ (richtig) bzw. „Es ist sicher, dass die Impfung nicht zu Gesichtslähmungen führt“ (falsch)). Ein einfacher Summen-Score addierte die Anzahl der richtigen Antworten (Summe von 0 bis 5). Unabhängig von der Präregistrierung ließen wir explorativ vor und nach der Informationspräsentation schätzen, wie viele von 1000 gegen Covid-19 geimpften (ungeimpften) Personen im Fall des Kontakts mit einem/r Infizierten erkranken würden.

### Analysen.

Gemäß unserer Präregistrierung unterschieden wir die Teilnehmenden nach ihrer Absicht, sich impfen zu lassen. Die Gruppen wurden wie folgt bezeichnet: Impfbereite, Impfgeneigte, Impfskeptische, Impfgegner:innen und Unentschiedene. Letztere unterschieden wir für unsere Hypothesen zusätzlich nach einer Angabe zu ihren Informationsbedürfnissen. Die Häufigkeiten der Gruppen, ohne Studienteilnehmende, die bereits geimpft waren, lassen sich Tabelle S2 im Onlinematerial entnehmen. Für die Analysen der präregistrierten Hypothesen wurden 2 Gruppen berücksichtigt: zum einen die Gruppe der Unentschiedenen mit Informationsbedürfnissen und Skeptischen, zum anderen die Gruppe der Unentschiedenen ohne Informationsbedürfnisse und Impfgeneigten. Einzelgruppenanalysen pro Präsentationsformat waren bis auf wenige Ausnahmen aufgrund der geringen Gruppengrößen nicht möglich.

Vor der Analyse der experimentellen Daten wurden etwa 20 % der Befragten ausgeschlossen (Abb. 1), die entweder das Kontrollitem zur Antwortsorgfalt („Diese Aussage dient der Qualitätssicherung: Bitte wählen Sie diese Antwort zusätzlich aus“) nicht korrekt beantwortet hatten oder mehr als das Fünffache der durchschnittlichen Bearbeitungszeit benötigten, Letztere unter der Annahme, dass sich die Personen wahrscheinlich anderen Aktivitäten zugewandt hatten.

Die Änderung der Gesamtabsicht wurde mit dem Wilcoxon-Test analysiert. Auch das Verstehen der Impfung wurde mit Rangtests analysiert. Die ANOVA mit wiederholten Messungen wurde für die Analysen der Einstellung und des Vertrauens in die mRNA-Impfstoffe durchgeführt. Alle Analysen wurden mit gewichteten Daten durchgeführt.

## Ergebnisse

### Teilnehmende.

Gemäß ihrer Impfabsicht ließen sich gewichtet 992 Impfbereite, 235 Impfgeneigte, 154 Unentschiedene, 111 Skeptische, 200 Impfgegner*innen und 660 bereits geimpfte Teilnehmende unterscheiden.

### Impfabsichten.

**Abb. 2:**
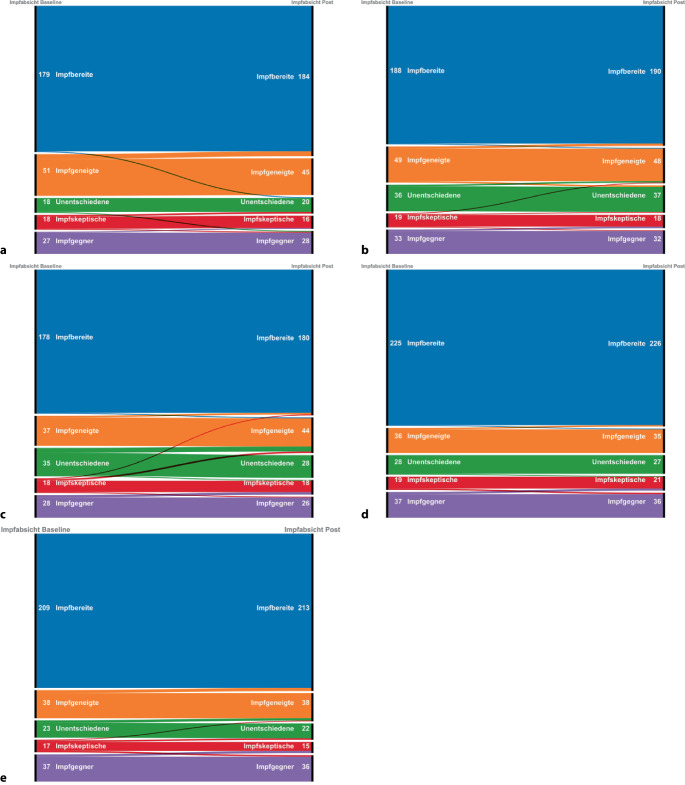
Veränderung der Anzahl von Teilnehmenden mit bestimmten Impfabsichten (von oben nach unten: Impfbereite, Impfgeneigte, Unentschiedene, Skeptische, Impfgegner*innen) von Baseline (links) nach Post (rechts) in Abhängigkeit der Informationspräsentationen: Werbung (**a**), tabellarische Faktenbox (**b**), grafische Faktenbox (**c**), Norm-Nudge (**d**), moralischer Nudge (**e**)

Unter den Ungeimpften veränderten sich (Abb. 2) die Impfabsichten weder in der Kontrollbedingung durch Werbung (Wilcoxon z = 1,09, *p* = 0,276), durch den Norm-Nudge (z = 1,00, *p* = 0,317) noch durch die Faktenboxtabelle (z = 1,79, *p* = 0,074). Die grafische Faktenbox (z = 2,59, *p* = 0,009) und der moralische Nudge hingegen erhöhten die Impfabsicht (z = 3,52, *p* < 0,001). Insgesamt gingen 12 % der Unentschiedenen und 8 % der Skeptischen zu einer verglichen mit vorher eher befürwortenden bzw. weniger ablehnenden Absicht über, wenn ihnen die grafische Faktenbox dargeboten wurde (Abb. 2c).

Unter den Unentschiedenen berichteten diejenigen mit Informationsbedürfnissen (Wilcoxon *n* = 37, z = 2,00, *p* = 0,046) stärkere Impfabsichten, wenn ihnen eine grafische Faktenbox präsentiert wurde. Dieser Effekt konnte für die tabellarische Faktenbox jedoch nicht bestätigt werden (*n* = 34, z = 1,73, *p* = 0,083), auch nicht für die anderen Präsentationsformate. Wobei aufgrund der geringen Stichprobe (*n* = 13 bis 26) das Aufdecken möglicher Effekte, so vorhanden, unwahrscheinlich war. Die Stichprobe der Unentschiedenen ohne Informationsbedürfnisse (alle Präsentationsbedingungen zusammen: *n* = 29) war ebenfalls zu klein für einen robusten Vergleich pro Bedingung.

Die kombinierte Analyse der Unentschiedenen mit Informationsbedürfnissen und Skeptischen bestätigte die Primärhypothese teilweise: Die Präsentation von grafischen Faktenboxen verstärkte die Impfabsicht (*n* = 58, z = 2,16, *p* = 0,031). Dies konnte jedoch für kein weiteres Präsentationsformat bestätigt werden.

Unter den Impfgeneigten und den Unentschiedenen ohne Informationsbedürfnisse führte nicht der Norm-Nudge (z = 0,58, *p* = 0,564), aber der moralische Nudge (z = 2,24, *p* = 0,025) eher zu definitiver Impfabsicht und bestätigte unsere Hypothese teilweise. Die Faktenboxen hatten keinen Effekt auf diese Gruppe.

Der Einfluss der Informationsbedürfnisse wurde mit Blick auf die Stichprobengrößen über alle Impfgruppen zusätzlich mit einer logistischen Regression untersucht, deren Zielvariable eine Veränderung in Richtung der Impfbereitschaft auswies. Informationsbedürfnisse zu haben erhöhte im Fall der grafischen Faktenbox (Gesamtmodell χ^2^(1) = 5,86, *p* = 0,015, Nagelkerke R^2^ = 0,06) die Chancen für eine solche Veränderung um den Faktor 4,76 (95 % Konfidenzintervall [1, 12, 19, 48], *p* = 0,034).

### Verstehen der Impfung (Comprehension).

**Abb. 3:**
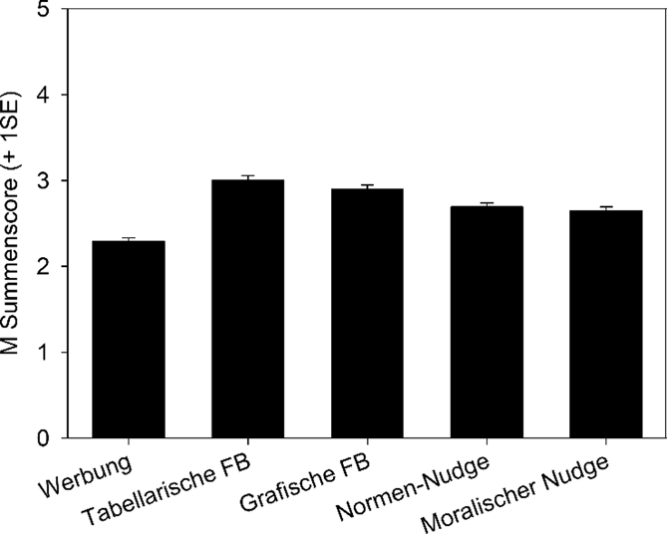
Mittlere Summenscores des Verstehens der Impfung in Abhängigkeit der Präsentationsbedingungen. Die Fehlerbalken zeigen die Standardfehler des Mittelwerts

Die Summenscores der ungeimpften Teilnehmenden variierten zwischen den Formaten (Kruskal-Wallis H(4) = 73,53, *p* < 0,001), mit den meisten korrekten Antworten unter jenen, denen die Faktenboxen vorlagen (Abb. 3). Verglichen mit der Kontrollbedingung (Werbung) zeigte sich bei der Gruppe der Unentschiedenen mit Informationsbedürfnissen und Skeptischen eine Verbesserung des Verständnisses durch Faktenboxen (*n* = 153, z = 1,98, *p* = 0,048). Ein vergleichbarer Effekt trat in der Gruppe der Impfgeneigten und Unentschiedenen ohne Informationsbedürfnisse auf (*n* = 174, z = 2,16, *p* = 0,031).

Unabhängig von der Präregistrierung wurde explorativ untersucht, ob sich die Einschätzungen des Basisrisikos für Ungeimpfte bzw. des reduzierten Risikos für gegen Covid-19-Geimpfte – jeweils im Fall eines Kontakts mit einer infizierten Person – durch die Informationspräsentation einer Faktenbox im Vergleich zu anderen Formaten ohne diese Information verbessern (Tabelle S2). Messwiederholte ANOVA-Berechnungen über alle Teilnehmenden bestätigten diese Interaktion von Präsentation und Messzeitpunkt auf die Einschätzungen des Basis- (F(4;2451) = 52,75, *p* < 0,001, η_p_^2^ = 0,08) und des reduzierten Risikos (F(4;2451) = 52,75, *p* < 0,001, η_p_^2^ = 0,08).

### Einstellung gegenüber den Impfstoffen.

Mit Blick auf die Gesamtstichprobe veränderte weder die Präsentation der tabellarischen (F(1;972) = 0,11, *p* = 0,744) noch der grafischen Faktenbox (F(1;937) < 0,01, *p* = 0,976) die Bewertung des Nutzen-Schaden-Verhältnisses der mRNA-Impfung relativ zu den Effekten der Kontrollbedingung. Das bedeutet, die Faktenboxen hatten keinen weniger positiven oder negativen Effekt auf die Bewertung des Nutzen-Schaden-Verhältnisses als Werbung. Dies galt auch für den Normanreiz (F(1;982) = 1,18, *p* = 0,277) und den moralischen Druck (F(1;970) = 1,61, *p* = 0,204).

Unter den Impfgeneigten hatten ebenfalls weder Norm- (F(1;95) = 1,43, *p* = 0,235) noch moralischer Nudge (F(1;101) = 3,32, *p* = 0,072) einen marginalen Bewertungseffekt über die Kontrollbedingung mit der Werbeanzeige hinaus. Und unter den Unentschiedenen bzw. den Skeptischen hatten weder die tabellarischen (F(1;64) = 0,09, *p* = 0,762) bzw. (F(1;45) = 1,18, *p* = 0,284) noch die grafischen Faktenboxen (F(1;58) = 0,21, *p* = 0,846) bzw. (F(1;40) = 3,04, *p* = 0,065) einen Einfluss, der von der Kontrollbedingung abwich.

**Vertrauen** in die Impfstoffe. Weder die Präsentationen der tabellarischen (F(1;972) = 0,31, *p* = 0,580) noch der grafischen Faktenbox (F(1;937) < 0,87, *p* = 0,352) veränderten das Vertrauen in die mRNA-Impfstoffe relativ zur Kontrollbedingung. Dies galt auch für den Norm-Anreiz (F(1;982) = 1,51, *p* = 0,220) und den moralischen Druck (F(1;970) = 2,13, *p* = 0,144). Tatsächlich stieg über alle Bedingungen hinweg das Vertrauen in die mRNA-Impfstoffe mit der Wiederholung der Frage (F(1;2455) = 64,20, *p* < 0,001, η_p_^2^ = 0,03). Ein ähnlicher Effekt war zudem auch für die Vektorimpfung zu beobachten (F(1;2455) = 38,33, *p* < 0,001, η_p_^2^ = 0,02).

Auch unter den Unentschiedenen und Skeptischen konnten tabellarische (F(1;64) = 0,51, *p* = 0,477) bzw. (F(1;45) = 0,89, *p* = 0,352) und grafische Faktenboxen (F(1;58) = 0,54, *p* = 0,467) bzw. (F(1;40) = 1,50, *p* = 0,229) das Vertrauen in die Impfstoffe gegenüber der Kontrollbedingung nicht erhöhen.

## Diskussion

Die Präsentation von grafischen Faktenboxen konnte insbesondere bei Unentschiedenen mit Informationsbedürfnissen und Skeptischen zum Verständnis der Impfung beitragen und deren Covid-19-Impfabsicht fördern. Dieser Effekt konnte für die tabellarische Fassung nicht gezeigt werden. Es kann jedoch nicht ausgeschlossen werden, dass aufgrund der Gruppengrößen und der Herausforderung, durch eine einmalige Informationspräsentation innerhalb eines Surveys eine Intention zu verändern, ein kleinerer Faktenboxeffekt bei der tabellarischen Fassung unerkannt blieb.

Evidenzbasierte behördliche Kommunikationsformate, die informiertes Entscheiden stärken, wirken der höchsten gesellschaftlichen Präferenz für hohe Impfraten in der Bevölkerung – erschließt man diese Präferenz aus dem Handeln des Bundesgesundheitsministeriums während der Pandemie, dem Auftrag der zuständigen Behörden sowie der Bevölkerungsunterstützung des Impfens – zumindest nicht entgegen. Darauf gab es bisher nur Hinweise durch eine Studie, die zeigte, dass das Nutzen-Schaden-Verhältnis einer Covid-19-Impfung durch die Präsentation der ersten Fassung der Faktenboxen nicht schlechter bewertet wird [30]. Aus den vorliegenden Ergebnissen lässt sich nun schließen, dass die vom Robert Koch-Institut im Jahr 2021 implementierten grafischen Faktenboxen informiertes Entscheiden bezüglich der Covid-19-Impfung sowie begründete Impfintentionen in relevanten Gruppen in Deutschland unterstützen konnten.

Zudem wurden sowohl die Einstellung als auch das Vertrauen in die Impfstoffe von den Faktenboxen im Vergleich zur Werbung nicht negativ beeinflusst. Die behördliche Aufklärung war also den werblichen Formaten diesbezüglich auch nicht unterlegen. In Kombination mit der Evidenzlage zum Wissenserwerb und informierten Entscheiden durch Faktenboxen [24, 29–32] und mit der Integrierbarkeit in verschiedenen Versorgungs- bzw. Lebenssituationen [54, 55] sowie Medienkanälen (Print, Online) liegt die Schlussfolgerung nahe, Faktenboxen als Kommunikationsformat im Repertoire der behördlichen Aufklärung zu berücksichtigen. Die Erkenntnisse dieser Studie legen jedoch nahe, dass der Nutzen von grafischen (mit Einfluss auf die Impfabsicht) und tabellarischen Formaten (ohne Einfluss auf Impfabsicht) je nach Versorgungssituation und Zielgruppe – etwa in Abhängigkeit vom Zahlenverständnis (Numeracy) – durchaus divergieren wird.

Der Effekt der evidenzbasierten grafischen Informationen auf die Impfabsichten hängt dieser Studie zufolge vom expliziten Bedürfnis nach Impfinformationen ab, wie anhand der Skeptischen deutlich wurde. Dies wirft die grundsätzliche Frage auf, ob Aufklärung – insbesondere durch die Förderung von Gesundheitskompetenz [56] – nicht zunächst dazu beitragen sollte, Informationsbedürfnisse überhaupt bewusst zu machen und eine reflektierte Auseinandersetzung darüber anzustoßen, welche Informationen für eine fundierte Impfentscheidung tatsächlich benötigt werden (Stichwort Informationsbedarfe). Hierzu wären Interventionsstudien mit wenig- bis nichtgesundheitskompetenten Menschen erforderlich. Doch selbst unter dem Gesichtspunkt der Deckung von Informationsbedarfen gilt: Evidenzbasierte Informationen können und sollten nicht als Instrument wissensunabhängiger Verhaltenssteuerung eingesetzt werden, um Impfbereitschaft oder Impfquoten zu erhöhen. So können beispielsweise sozial benachteiligte Menschen bestimmte Verhaltensbotschaften aufgrund fehlender Ressourcen kaum umsetzen mit dem Risiko, dadurch zusätzlich stigmatisiert und diskriminiert zu werden. Effektiv implementierte Prävention, die dazu geeignet ist, ungleiche Gesundheitschancen zu minimieren, ist auf Verhältnisveränderungen angewiesen, einschließlich der sozialen, kulturellen, technisch-materiellen sowie ökologischen Lebensbedingungen [57].

Im Einklang mit der Literatur zur Impfverhaltenssteuerung über das Schutzargument der Gemeinschaft, um die prosoziale Impfung zu erhöhen [58], verstärkte moralischer Druck die Covid-19-Impfbereitschaft, insbesondere unter Impfgeneigten und Unentschiedenen ohne Informationsbedürfnisse. Somit sind solche Nudges gerade für diese Gruppe ein wirksames Mittel zur Verhaltenssteuerung, welches zudem im Rahmen der Studie nicht das Vertrauen in die Impfstoffe unterminierte. Mit Blick auf ihre Integration in eine Impfkampagne ist zusätzlich zu berücksichtigen, dass Entwickler solcher Nudges ein ausreichendes „Verständnis der Verhaltensmechanismen“ [52] sowie der ethischen Implikationen haben sollten [59], um Nebeneffekte (z. B. nichtintendierte Impfzurückhaltung, Vertrauensverluste gegenüber dem Gesundheitssystem) zu vermeiden. Darüber hinaus führte das Norm-Nudging, welches die regulatorisch geplanten Gruppenunterschiede zwischen Geimpften und Ungeimpften verdeutlichte, nicht zu einer entsprechenden Veränderung der Absichten. Dies steht im Gegensatz zu anderen Ergebnissen [16], ließe sich aber möglicherweise auf Limitationen unserer Studie zurückführen.

### Limitationen.

Die Ergebnisse des direkten Vergleichs zwischen Nudges und Faktenboxen sind insgesamt nur eingeschränkt interpretierbar. Ein Grund dafür liegt darin, dass sich beide nicht nur in ihrer Zielstellung – Verhaltenssteuerung versus Informationsvermittlung – unterscheiden, sondern auch deutlich in ihrer informationellen Ausgestaltung. Denkbar wären jedoch alternative Formate, bei denen Nudges um Faktenboxinformationen ergänzt werden. Ein weiterer Grund liegt darin, dass die Nudges selbst nicht pilotiert und validiert wurden. Die Konsequenz zeigt sich exemplarisch am Norm-Nudge: Statt zur beabsichtigten Reflexion der Teilnehmenden über ihre relative Position zur geltenden Norm anzuregen, stand mutmaßlich der antizipierte persönliche Nutzen der angekündigten Freiheiten für Geimpfte im Vordergrund. Alternativ wären Nudges vorstellbar, die infolge strukturierter Pilotierung und Validierung letztlich stärkere Verhaltenseffekte (z. B. auf die Impfabsicht) erzielen könnten.

Des Weiteren war die Wahl der Kontrollgruppe mit Nachteilen verbunden, da Werbung naturgemäß Einfluss auf Absichten, Einstellungen und Vertrauen nehmen kann. Dies erschwerte die Interpretation von Unterschieden gegenüber den Interventionsbedingungen. Hier stand jedoch die Berücksichtigung einer ökologisch validen Vergleichsbedingung im Vordergrund.

Drittens kann auch ein Einfluss von thematischem Priming nicht ausgeschlossen werden, denn z. B. wurde im vorhergehenden Itemblock nach den Einstellungen zur Corona-Politik gefragt. Diese waren in der Ausnahmesituation der fortgeschrittenen Pandemie bereits stark polarisiert. Das heißt, sehr festgelegte Einstellungen (z. B. stark negativ gegenüber dem Corona-Management oder stark negativ gegenüber jenen, die sich dem Corona-Management nicht anschlossen) konnten die Folgefragen, die sich auf das Impfen bezogen, insoweit beeinflussen, dass Variationen durch die experimentellen Stimuli erschwert wurden.

Kritisch ist der Einsatz der Itemkonstruktion zur Messung des Verständnisses des mRNA-Impfstoffs einzuschätzen, welche nur unter Wissenschaftler*innen im Team diskutiert, aber nicht validiert wurde. Ein Hindernis für die Entwicklung von validen Wissenstests bzw. Testbatterien ist jedoch die sich kontinuierlich ändernde Evidenzlage zu Nutzen, Schaden und epistemischer Unsicherheit in einer Pandemie. Dadurch wären kontinuierliche Überarbeitungen solcher Wissenstests erforderlich, die kaum bis zum nächsten Evidenz-Update zu validieren wären.

Das Verstehen der Impfung wurde ausschließlich auf Basis von Informationspräsentationen erfasst. Es ist jedoch nicht davon auszugehen, dass sich das Vorwissen trotz Randomisierung rein zufällig absolut gleichmäßig auf die Gruppen verteilt hat. Eine Baseline-Messung wäre für weitere Studien wie diese vorzuziehen, weil dann für Vorwissen effektiv kontrolliert werden könnte. Damit verbunden ist auch die Bedeutung größerer Teilstichproben mithilfe weniger breit angelegter Designs, denn die erreichten Gruppengrößen innerhalb der Präsentationsbedingungen machten die Aufdeckung vorhandener moderater und vor allem kleiner Effekte weniger wahrscheinlich.

## Fazit

Angesichts spezifischer unentschiedener und eher skeptischer Personen, von denen viele ein Informationsbedürfnis haben, das häufig nicht adäquat adressiert wird, ist es eine Aufgabe behördlicher Impfkampagnen, zunächst die Bedürfnisse systematisch zu erfassen und darauf aufbauend zwischen objektivem und individuellem Informationsbedarf zu differenzieren, um beides zielgenau adressieren zu können. Obwohl die Impfkampagne in Deutschland stark auf werbliche Elemente setzte, wurde mit der Implementierung evidenzbasierter Faktenboxen durch das RKI und das Harding-Zentrum für Risikokompetenz während der Covid-19-Pandemie im Jahr 2021 ein relevanter Beitrag geleistet, um insbesondere Personen mit Informationsbedürfnissen und Zweifeln gezielt zu unterstützten.

## Supplementary Information

Der Anhang enthält die verwendeten Originalstimuli der Präsentationsbedingungen des Experiments, den Fragebogen sowie die deskriptiven Statistiken der Risikoeinschätzungen inklusive der Fallzahlen nach Bedingungen und Impfabsichten.

## Data Availability

Die während der vorliegenden Studie erzeugten und analysierten Datensätze sind auf begründete Anfrage bei der Korrespondenzperson erhältlich.

## Footnotes

1 Präregistrierung siehe https://aspredicted.org/DDI_WHT.
