# Supplementary material for: Enlightenment through official vaccination communication: an experimental study comparing evidence-based fact boxes with nudges and advertising
Source: Bundesgesundheitsblatt Gesundheitsforschung Gesundheitsschutz. 2025 Aug 4;68(9):1024–34. [Article in German] doi: 10.1007/s00103-025-04109-2 (PMC12391170; doi:10.1007/s00103-025-04109-2)
Supplement: Supplementary file 1 — Der Anhang enthält die verwendeten Originalstimuli der Präsentationsbedingungen des Experiments, den Fragebogen sowie die deskriptiven Statistiken der Risikoeinschätzungen inklusive der Fallzahlen nach Bedingungen und Impfabsichten. [file 103_2025_4109_MOESM1_ESM.pdf]

Onlinematerial

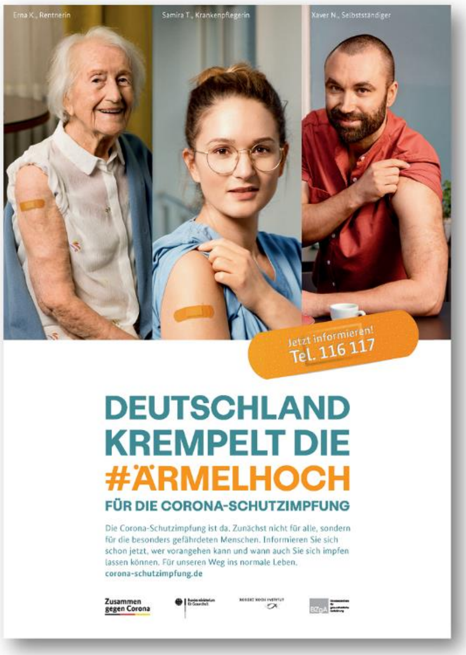

Abbildung S1. Präsentierte Information einer Werbeanzeige der Bundesregierung („Ärmel hoch“) in der Kontrollbedingung. Quelle: Bundesministerium der Gesundheit, abgerufen unter https://www.spiegel.de/politik/deutschland/corona-krise-bundesregierung-startet-werbekampagne-fuer-corona-impfungen-a-6568fac8-1e8d-489c-948a-6e85433c789c (Letzter Zugriff, 14. Mai 2025)

A

Faktenbox: Wie **sicher** und **wirksam** sind mRNA-Schutzimpfungen gegen COVID-19 für Erwachsene unter 60 Jahren?

HARDING-ZENTRUM FÜR RISIKOKOMPETENZ — ROBERT KOCH INSTITUT

Diese Faktenbox vergleicht Erwachsene unter 60 Jahren ohne Impfung gegen COVID-19 (linke Seite) mit geimpften Erwachsenen (rechte Seite).
Es wird angenommen, dass 240 von 1.000 Nichtgeimpften erkranken. Dies ist mit Ihrem Risiko vergleichbar, bei engem Kontakt mit einem/r Infizierten zu erkranken.

	Von 1.000 **nicht geimpften** Erwachsenen	Von 1.000 **geimpften** Erwachsenen
**Nutzen der Impfung**		
Wie viele **erkranken** an COVID-19?	240	10
… und müssen – je nach Alter oder Vorerkrankung – aufgrund eines schweren Verlaufs **im Krankenhaus behandelt werden**?	6 bis 31	0 bis 1
… und leiden dauerhaft an den **Folgen einer COVID-19-Erkrankung**?	Die Zahlen sind noch unsicher. Es gibt deutliche Hinweise auf dauerhafte Atemnot und Gedächtnisstörungen durch COVID-19 („Long-COVID“).	
**Schaden durch die Impfung**		
Wie viele können aufgrund einer Impfdosis an einzelnen darauffolgenden Tagen nicht an ihrem Alltag teilnehmen (wegen **vorübergehender Erschöpfung, Fieber, Schmerzen oder Schüttelfrost**)?	0	82
Wie viele erleiden aufgrund einer Impfdosis innerhalb eines Monats einen **schweren Schaden** (z. B. allergische Überreaktion)?	0	Annähernd 0
Wie viele erleiden aufgrund der Impfung einen **dauerhaften Schaden**?	0	Es gibt derzeit keine Hinweise auf dauerhafte Schäden.

Hinweis: Typische Impfreaktionen, die den Arm oder den ganzen Körper betreffen können, klingen in der Regel nach ein bis zwei Tagen ab. Das Auftreten seltener Impfreaktionen wie z. B. allergische Überreaktionen sowie mögliche Zusammenhänge der Impfung mit untypischen Reaktionen (z. B. Schlaflosigkeit, vergrößerte Lymphknoten und vorübergehende Gesichtslähmungen) werden zurzeit untersucht. Es wird noch erforscht, wie lange der Impfschutz anhält.

Letztes Update: *14. April 2021 | Zu den Studien und offenen Fragen sowie zur aktuellen Version: www.hardingcenter.de/de/faktenboxen*
Harding-Zentrum für Risikokompetenz (Fakultät für Gesundheitswissenschaften Brandenburg, Universität Potsdam), Robert Koch-Institut

Quellen *zu den Impfstoffen Comirnaty (Hersteller BioNTech/Pfizer) und Moderna (Hersteller Moderna): Baden 2020. NEJM; BioNTech & Pfizer 2020. www.comirnatyeducation.de; CDC 2021. MMWR; EMA 2020. www.ema.europa.eu; FDA 2020. FDA Briefing Document; Polack 2020. NEJM; RKI 2020. Meldedaten; STIKO 2021. Epidemiologisches Bulletin.*

Wissenschaftskommunikation Robert Koch-Institut wiko@rki.de

B

Faktenbox: Wie **sicher** und **wirksam** sind mRNA-Schutzimpfungen gegen COVID-19 für Erwachsene ab 60 Jahren?

HARDING-ZENTRUM FÜR RISIKOKOMPETENZ — ROBERT KOCH INSTITUT

Diese Faktenbox vergleicht Erwachsene ab etwa 60 Jahren ohne Impfung gegen COVID-19 (linke Seite) mit geimpften Erwachsenen (rechte Seite).
Es wird angenommen, dass 240 von 1.000 Nichtgeimpften erkranken. Dies ist mit Ihrem Risiko vergleichbar, bei engem Kontakt mit einem/r Infizierten zu erkranken.

	Von 1.000 **nicht geimpften** Erwachsenen	Von 1.000 **geimpften** Erwachsenen
**Nutzen der Impfung**		
Wie viele **erkranken** an COVID-19?	240	24
… und müssen – je nach Alter oder Vorerkrankung – aufgrund eines schweren Verlaufs **im Krankenhaus behandelt werden**?	36 bis 120	1 bis 5
… und leiden dauerhaft an den **Folgen einer COVID-19-Erkrankung**?	Die Zahlen sind noch unsicher. Es gibt deutliche Hinweise auf dauerhafte Atemnot und Gedächtnisstörungen durch COVID-19 („Long-COVID“).	
**Schaden durch die Impfung**		
Wie viele können aufgrund einer Impfdosis an einzelnen darauffolgenden Tagen nicht an ihrem Alltag teilnehmen (wegen **vorübergehender Erschöpfung, Fieber, Schmerzen oder Schüttelfrost**)?	0	49
Wie viele erleiden aufgrund einer Impfdosis innerhalb eines Monats einen **schweren Schaden** (z. B. allergische Überreaktion)?	0	Annähernd 0
Wie viele erleiden aufgrund der Impfung einen **dauerhaften Schaden**?	0	Es gibt derzeit keine Hinweise auf dauerhafte Schäden.

Hinweis: Typische Impfreaktionen, die den Arm oder den ganzen Körper betreffen können, klingen in der Regel nach ein bis zwei Tagen ab. Das Auftreten seltener Impfreaktionen wie z. B. allergische Überreaktionen sowie mögliche Zusammenhänge der Impfung mit untypischen Reaktionen (z. B. Schlaflosigkeit, vergrößerte Lymphknoten und vorübergehende Gesichtslähmungen) werden zurzeit untersucht. Es wird noch erforscht, wie lange der Impfschutz anhält.

Letztes Update: *14. April 2021 | Zu den Studien und offenen Fragen sowie zur aktuellen Version: www.hardingcenter.de/de/faktenboxen*
Harding-Zentrum für Risikokompetenz (Fakultät für Gesundheitswissenschaften Brandenburg, Universität Potsdam), Robert Koch-Institut

Quellen *zu den Impfstoffen Comirnaty (Hersteller BioNTech/Pfizer) und Moderna (Hersteller Moderna): Baden 2020. NEJM; BioNTech & Pfizer 2020. www.comirnatyeducation.de; CDC 2021. MMWR; EMA 2020. www.ema.europa.eu; FDA 2020. FDA Briefing Document; Polack 2020. NEJM; RKI 2020. Meldedaten; STIKO 2021. Epidemiologisches Bulletin.*

Wissenschaftskommunikation Robert Koch-Institut wiko@rki.de

Abbildungen S2A-B. Präsentierte Information in Form einer tabellarischen RKI-Faktenbox für Menschen unter (A) und ab (B) 60 Jahren. Alle Faktenboxen des Harding-Zentrum für Risikokompetenz und das dazugehörige Begleitmaterial sind lizenziert unter einer Creative Commons BY-NC-ND 4.0 Lizenz.

A

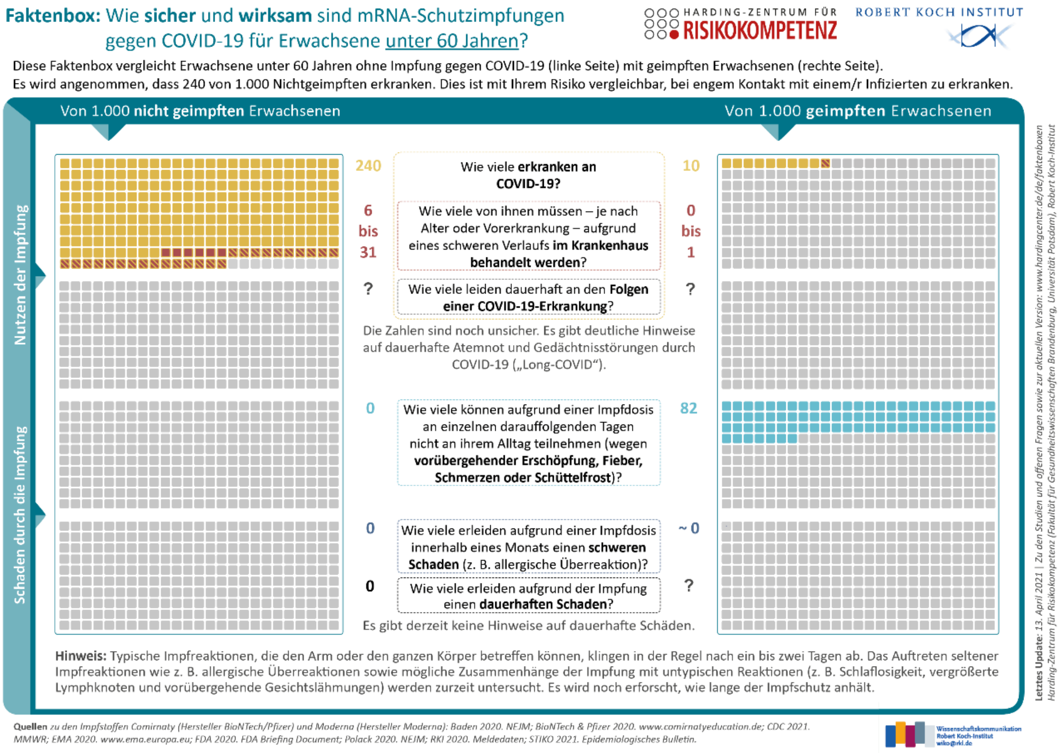

B

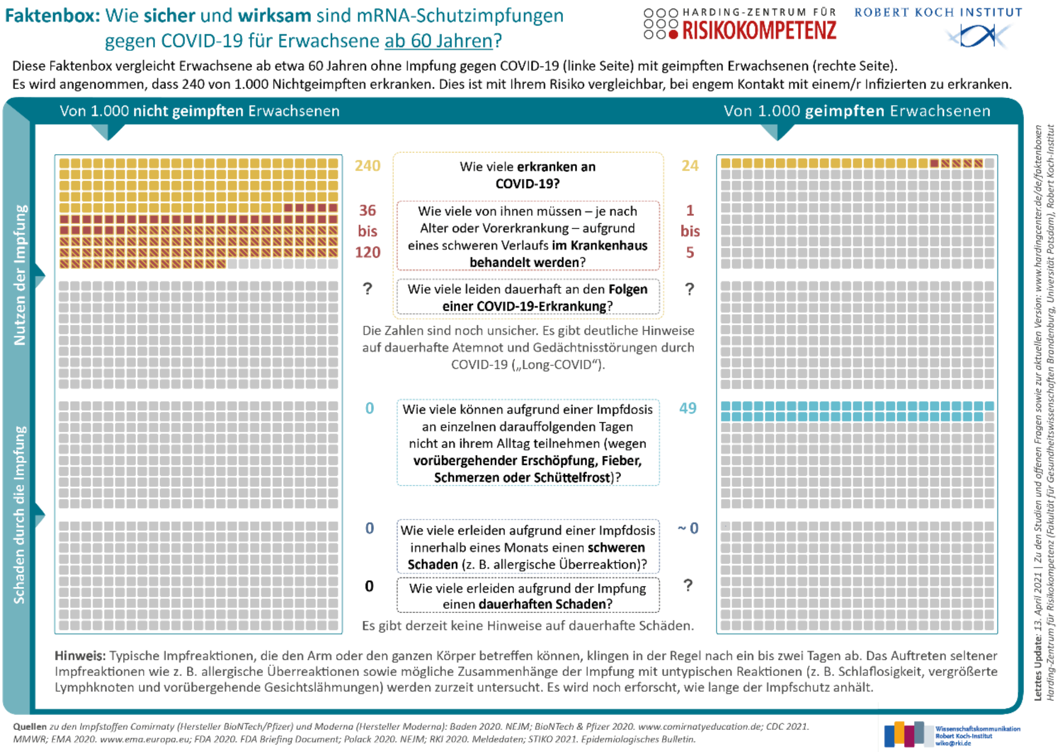

Abbildungen S3A-B. Präsentierte Information in Form einer grafischen RKI-Faktenbox für Menschen unter (A) und ab (B) 60 Jahren. Quelle: Alle Faktenboxen des Harding-Zentrum für Risikokompetenz und das dazugehörige Begleitmaterial sind lizenziert unter einer Creative Commons BY-NC-ND 4.0 Lizenz.

„Corona-Erleichterungen für vollständig Geimpfte in Sicht“

- Vorschläge der Bundesregierung zu Erleichterungen:
  - keine Kontaktbeschränkungen mehr: Großeltern könnten ihre Enkel zum Beispiel wieder gemeinsam besuchen, wenn sie geimpft sind. Selbst Geimpften-Partys wären möglich.
  - Außengastronomie: Zwangstests und Beschränkungen entfallen [bild.de, 23.04.21]
  - Keine Zwangstests mehr vor Reiseantritt
  - Keine Quarantäne mehr nach Reiserückkehr
  - Keine Testpflicht mehr in Läden und Märkten, Kultureinrichtungen, körpernahen Dienstleistungen und Sport [fr.de, 27.04.21]
- Am 28. Mai soll der Bundesrat entscheiden, welche Rechte Geimpfte künftig haben sollen
- Bayern, Rheinland-Pfalz und Hessen haben bereits ihre Verordnungen zugunsten der Geimpften angepasst [merkur.de, 28.04.21]

Abbildung S4. Präsentierte Information in der Bedingung mit der Norm-Ankündigung (nudge). Quelle: Harding-Zentrum für Risikokompetenz, keine frühere Veröffentlichung.

„Am Ende hilft nur eine Impfpflicht“

- Impfen ist historisch gesehen die mit Abstand erfolgreichste Waffe zur Besserung der Gesundheit der Gesellschaft. Umso mehr erstaunt, dass die Impfbereitschaft weltweit zurückgeht

- Epidemiologen: eine Pandemie ist erst bei "Herdenimmunität" bekämpft: 80 Prozent der Menschen müssen gegen COVID-19 geimpft oder davon genesen sein; geringe Impfbereitschaft verhindert Herdenimmunität

- Die Minderheit der Impfskeptiker schädigt die Gesundung der Gemeinschaft. Die Freiheit der Gegner bedroht die Freiheit aller:
  - Impfverweigerung als unkooperatives, antisoziales und zutiefst eigennütziges Verhalten
  - Impfverweigerung wie andere Formen mangelnder Kooperation gesellschaftlich zu ächten, seien es Korruption, Lügen oder die Verschmutzung der Umwelt
  - Impfverweigerung ohne triftigen Grund als Trittbrettfahren der übelsten Sorte [faz.net einschließlich Interviewzitate, 20.02.21]

Abbildung S5. Präsentierte Information in der Bedingung mit moralischem Druck (nudge). Quelle: Harding-Zentrum für Risikokompetenz, keine frühere Veröffentlichung.

Tabelle S1. Fragebogen Experiment in CoronaCOMPASS Welle 11

63.	[Impfabsicht]
Inzwischen stehen Impfstoffe gegen das Corona-Virus zur Verfügung. Wenn Sie die Möglichkeit erhalten, werden Sie sich dann gegen Corona impfen lassen?	
(1)	Ja, sicher
(2)	Ja, wahrscheinlich
(3)	Nein, wahrscheinlich nicht
(4)	Nein, ganz sicher nicht
(5)	Kann ich noch nicht einschätzen / bin noch unentschieden
(6)	Bin bereits geimpft ⍰ F63a/c
63a.	[Geimpfte: Impfstoff bei Erstimpfung]
Mit welchem Impfstoff wurden Sie bei der Erstimpfung geimpft?	
(1)	BioNTech-Pfizer
(2)	Moderna
(3)	AstraZeneca
(4)	Mit einem anderen Impfstoff, nämlich: __________ [offene Texteingabe]
(5)	Weiß nicht
63c.	[Geimpfte: Zeitpunkt der Erstimpfung]
Wann (ungefähr) wurden Sie erstmals geimpft?	
(1)	Vor Dezember 2020
(2)	Dezember 2020
(3)	Januar 2021
(4)	Februar 2021
(5)	März 2021
(6)	April 2021
(7)	Weiß nicht
71a.	[Impfen – Verhältnis Nutzen - Risiko]
Wie schätzen Sie für sich persönlich das Verhältnis von möglichem Nutzen und Risiko der Corona-Impfung ein?	
Verwenden Sie dazu bitte eine Skala von 0 bis 10. Der Wert 0 bedeutet, das Risiko überwiegt klar den möglichen Nutzen. Der Wert 10 bedeutet, der mögliche Nutzen überwiegt klar das Risiko. Mit den Werten dazwischen können Sie Ihre Einschätzung abstufen.	
(1)	In Bezug auf mRNA-Impfungen (z.B. Impfstoffe von Biontech-Pfizer und Moderna)

Das Risiko überwiegt klar den möglichen Nutzen										Der mögliche Nutzen überwiegt klar das Risiko
0	1	2	3	4	5	6	7	8	9	10

(2) In Bezug auf Vektor-Impfungen (z.B. Impfstoffe von AstraZeneca und Johnson&Johnson)

Das Risiko überwiegt klar den möglichen Nutzen										Der mögliche Nutzen überwiegt klar das Risiko
0	1	2	3	4	5	6	7	8	9	10

710. [Impfstoff-Vertrauen]

Wie groß ist Ihr Vertrauen in die Corona-Impfstoffe?

Verwenden Sie dazu bitte eine Skala von 0 bis 10. Der Wert 0 bedeutet, überhaupt kein Vertrauen. Der Wert 10 bedeutet, vollstes Vertrauen. Mit den Werten dazwischen können Sie Ihre Einschätzung abstufen.

(1) In Bezug auf mRNA-Impfstoffe (z.B. Impfstoffe von Biontech-Pfizer und Moderna)

Überhaupt kein Vertrauen									Vollstes Vertrauen	
0	1	2	3	4	5	6	7	8	9	10

(2) In Bezug auf Vektor-Impfstoffe (z.B. Impfstoffe von AstraZeneca und Johnson&Johnson)

Überhaupt kein Vertrauen										Vollstes Vertrauen
0	1	2	3	4	5	6	7	8	9	10

712. [Informationsbedürfnis mRNA-Impfung]

Im Folgenden geht es nur noch um die sogenannte mRNA-Impfung, also die Impfstoffe von Biontech-Pfizer und von Moderna.

Unsere erste Frage: Welche Informationen zur mRNA-Impfung hätten Sie gern?

Bitte kreuzen Sie alle zutreffenden Antworten an.

Ich hätte gern Informationen zur Wirksamkeit der mRNA-Impfung

Ich hätte gern Informationen zur Sicherheit der mRNA-Impfung

Ich hätte gern Informationen zu den offenen Fragen bezüglich der mRNA-Impfung

Ich brauche keine der genannten Informationen

74. [Impfen – Basisrisiko - Baseline]

Nun möchten wir konkreter werden. Stellen Sie sich bitte 1.000 Menschen vor, die nicht gegen das Coronavirus geimpft wurden.

Was schätzen Sie, wie viele würden an COVID-19 erkranken, wenn sie in engen Kontakt mit einem Infizierten gekommen wären?

________ Menschen
75. [Impfen – Reduziertes Risiko Baseline]
Stellen Sie sich nun bitte 1.000 Menschen vor, die gegen das Coronavirus geimpft wurden.
Was schätzen Sie, wie viele würden an COVID-19 erkranken, wenn sie in engen Kontakt mit einem Infizierten gekommen wären?
________ Menschen
INTERVENTION
SPLIT A SPLIT B SPLIT C SPLIT D SPLIT E
76r. [Comprehension]
Darstellung: Mehrfachauswahl untereinander, keine Antwortpflicht, Frage unterhalb der Treatments auf derselben Seite darstellen
Filter: Keiner
Fragetext:
Welche der folgenden Aussagen sind Ihrer Ansicht nach richtig?
Erhält man eine mRNA-Schutzimpfung…
[random]
- …erhöht sich das Risiko deutlich, dass man in den Tagen nach einer Impfdosis zu erschöpft für den Alltag ist
- …hat man ein 1%iges Risiko (10 von 1.000 Menschen), aufgrund der Impfung einen schwerwiegenden Gesundheitsschaden zu erleiden
- …ist unsicher, ob die Impfung Spätfolgen nach sich zieht
- …diese Aussage dient der Qualitätssicherung: Bitte wählen Sie diese Antwort zusätzlich aus
- …ist sicher, dass sie nicht zu Lähmungen im Gesicht führt
- …schrumpft das Risiko etwa um den Faktor 20 bei Kontakt mit dem Coronavirus schwer an COVID-19 zu erkranken
[Ausleitungstext]
63i. [Wiederholung Impfabsicht für bereits Geimpfte]
Sie hatten angegeben, dass Sie bereits geimpft sind. Wenn Ihr Impfschutz voraussichtlich nur 1 Jahr lang anhält, werden Sie sich dann nächstes Jahr mit einer Auffrischungsimpfung wieder gegen Corona impfen lassen?
(1) Ja, sicher
(2) Ja, wahrscheinlich
(3) Nein, wahrscheinlich nicht
(4) Nein, ganz sicher nicht

(5) Kann ich noch nicht einschätzen / bin noch unentschieden

63w. [Wiederholung Impfabsicht für Noch nicht Geimpfte]

Noch einmal gefragt: Wenn Sie die Möglichkeit erhalten, werden Sie sich dann gegen Corona impfen lassen?

(1) Ja, sicher
(2) Ja, wahrscheinlich
(3) Nein, wahrscheinlich nicht
(4) Nein, ganz sicher nicht
(5) Kann ich noch nicht einschätzen / bin noch unentschieden

71w. [Wiederholung: Impfen – Verhältnis Nutzen - Risiko]

Noch einmal gefragt: Wie schätzen Sie für sich persönlich das Verhältnis von möglichem Nutzen und Risiko der Corona-Impfung ein?

Verwenden Sie dazu bitte eine Skala von 0 bis 10. Der Wert 0 bedeutet, das Risiko überwiegt klar den möglichen Nutzen. Der Wert 10 bedeutet, der mögliche Nutzen überwiegt klar das Risiko. Mit den Werten dazwischen können Sie Ihre Einschätzung abstufen.

(1) In Bezug auf mRNA-Impfungen (z.B. Impfstoffe von Biontech-Pfizer und Moderna)

Das Risiko überwiegt klar den möglichen Nutzen Der mögliche Nutzen überwiegt klar das Risiko

0 1 2 3 4 5 6 7 8 9 10

(2) In Bezug auf Vektor-Impfungen (z.B. Impfstoffe von AstraZeneca und Johnson&Johnson)

Das Risiko überwiegt klar den möglichen Nutzen Der mögliche Nutzen überwiegt klar das Risiko

0 1 2 3 4 5 6 7 8 9 10

710w. [Wiederholung Impfstoff-Vertrauen]

Wie groß ist Ihr Vertrauen in die Impfstoffe gegen COVID-19?

Verwenden Sie dazu bitte eine Skala von 0 bis 10. Der Wert 0 bedeutet, überhaupt kein Vertrauen. Der Wert 10 bedeutet, vollstes Vertrauen. Mit den Werten dazwischen können Sie Ihre Einschätzung abstufen.

(1) In Bezug auf mRNA-Impfstoffe (z.B. Impfstoffe von Biontech-Pfizer und Moderna)

Überhaupt kein Vertrauen Vollstes Vertrauen

0 1 2 3 4 5 6 7 8 9 10

(2) In Bezug auf Vektor-Impfstoffe (z.B. Impfstoffe von AstraZeneca und Johnson&Johnson)

Überhaupt kein Vertrauen Vollstes Vertrauen

0 1 2 3 4 5 6 7 8 9 10

74w. [Wiederholung: Impfen – Basisrisiko Post]
Stellen Sie sich nun bitte noch einmal 1.000 Menschen vor, die nicht gegen das Coronavirus geimpft wurden.
Was schätzen Sie, wie viele würden an COVID-19 erkranken, wenn sie in engen Kontakt mit einem Infizierten gekommen wären?
________ Menschen
75w. [Wiederholung: Impfen – Reduziertes Risiko Post]
Stellen Sie sich nun bitte noch einmal 1.000 Menschen vor, die gegen das Coronavirus geimpft wurden.
Was schätzen Sie, wie viele würden an COVID-19 erkranken, wenn sie in engen Kontakt mit einem Infizierten gekommen wären?
________ Menschen

Tabelle S2. Gruppenspezifische und formatspezifische Häufigkeiten (n) der ungeimpften Studienteilnehmenden sowie ihre Schätzungen des Basisrisikos und des Risikos von Geimpften in Fall eines Kontaktes zu erkranken, gemessen vor und nach Informationspräsentation.

Schätzung nach	Werbung			Tabellarische Faktenbox			Grafische Faktenbox			Norm-Nudge			Moralischer Nudge		
Gruppe		Baseline	Post		Baseline	Post		Baseline	Post		Baseline	Post		Baseline	Post
	n	M/SD	M/SD	n	M/SD	M/SD	n	M/SD	M/SD	n	M/SD	M/SD	n	M/SD	M/SD
Impfbereite															
Basisrisiko	177	533/365	557/351	200	518/341	325/250	184	502/360	350/293	224	522/354	519/358	207	512/351	530/348
Geimpft. Risiko	177	97/177	68/110	200	74/135	28/86	184	65/134	44/111	224	75/158	61/141	207	55/92	46/77
Impfgeneigte															
Basisrisiko	54	337/323	349/341	58	401/367	327/275	39	423/369	367/334	40	423/348	448/357	44	373/331	390/343
Geimpft. Risiko	54	85/140	80/131	58	90/145	79/181	39	143/235	103/227	40	121/196	99/191	44	59/86	46/65
Unentschiedene															
Basisrisiko	20	283/340	286/335	42	274/308	207/188	41	415/347	3477309	29	3547345	346/328	22	346/378	336/364
Geimpft. Risiko	20	103/215	109/214	42	137/222	43/107	41	179/224	103/140	29	1867252	174/249	22	138/246	124/212
Skeptische															
Basisrisiko	20	159/271	159/271	26	168/250	213/231	21	473/454	4127433	24	200/281	201/281	20	295/344	276/285
Geimpft. Risiko	20	124/240	124/240	26	75/147	54/139	21	272/343	234/336	24	23/27	20722	20	123/160	139/163
Impfgegner*in															
Basisrisiko	34	190/327	211/334	41	88/161	86/119	35	222/311	221/287	40	142/230	140/231	50	65/130	66/129
Geimpft. Risiko	34	194/312	2107316	41	73/169	31/59	35	160/271	144/244	40	104/205	109/211	50	47/96	49798

Hinweis: Teilnehmende, die zum Zeitpunkt der Studie bereits geimpft worden waren (n=660), wurden hier nicht berücksichtigt, da sie keiner der Gruppen zugerechnet werden konnten.
